# High-Pressure Synthesis of Non-Stoichiometric Li_*x*_WO_3_ (0.5 ≤ *x* ≤ 1.0) with LiNbO_3_ Structure

**DOI:** 10.3390/inorganics7050063

**Published:** 2019

**Authors:** Kohdai Ishida, Yuya Ikeuchi, Cédric Tassel, Hiroshi Takatsu, Craig M. Brown, Hiroshi Kageyama

**Affiliations:** 1 Department of Energy and Hydrocarbon Chemistry, Graduate School of Engineering, Kyoto University, Nishikyo-ku, Kyoto 615-8510, Japan;; 2 Center for Neutron Research, National Institute of Standards and Technology, Gaithersburg, MD 20899, USA;; 3 Department of Chemical and Biochemical Engineering, University of Delaware, Newark, DE 19716, USA

**Keywords:** high pressure, LiNbO_3_, LiWO_3_, tungsten bronze

## Abstract

Compounds with the LiNbO_3_-type structure are important for a variety of applications, such as piezoelectric sensors, while recent attention has been paid to magnetic and electronic properties. However, all the materials reported are stoichiometric. This work reports on the high-pressure synthesis of lithium tungsten bronze Li_*x*_WO_3_ with the LiNbO_3_-type structure, with a substantial non-stoichiometry (0.5 ≤ *x* ≤ 1). Li_0.8_WO_3_ exhibit a metallic conductivity. This phase is related to an ambient-pressure perovskite phase (0 ≤ *x* ≤ 0.5) by the octahedral tilting switching between a^−^a^−^a^−^ and a^+^a^+^a^+^.

## 1. Introduction

The lack of inversion symmetry in LiNbO_3_ allows ferroelectricity, piezoelectricity, pyroelectricity, and second-order nonlinear optical behavior [1,2]. These properties of LiNbO_3_ and related oxide insulators (e.g., LiTaO_3_ and ZnSnO_3_) lead to various industrial applications, such as waveguides, modulators, nonlinear crystals, and piezoelectric sensors. Recent research has added magnetic and electronic properties to this structural type. To obtain such materials with finite *d* electrons, the high-pressure synthesis approach was employed. ScFeO_3_ exhibits a magnetic ordering far above room temperature [3], while in MnTaO_2_N exhibits a strong bending of Mn–O–Mn angle, in the extended MnO_6_ network introduces spin frustration, resulting in a spiral spin order at low temperature [4]. LiOsO_3_ undergoes a structural phase transition from a centrosymmetric (*R*−3*c*) structure to a noncentrosymmetric (*R*3*c*) structure at 140K [5]. A “polar” metallicity in the low-temperature phase may offer an interesting platform for exotic electronic phases to control carrier density by, e.g., introducing anion/cation deficiency or substitution is essential. Unlike perovskites, however, all the known LiNbO_3_-type compounds are stoichiometric.

The present work stems from our recent studies on potassium and sodium tungsten bronzes, revealing the capability of high-pressure synthesis to increase the alkali-metal content [6,7]. For K_*x*_WO_3_, high pressure allows the formation of a stoichiometric tetragonal phase K_0.6_WO_3_ (K_3_W_5_O_15_) with anomalous metallic behavior and KWO_3_ with the ideal perovskite (*Pm*−3*m*) structure [6]. The high-pressure methodology also produces a stoichiometric NaWO_3_, in which a distorted perovskite (*Im*−3) structure gives rise to a novel rattling phenomenon [7]. From these results, it is clear that the high-pressure condition can suppress undesirable volatilization of alkali metals. The high compressibility of alkali metals may also allow for more incorporation of Na or K ions into the available lattice site.

**Figure 1.**
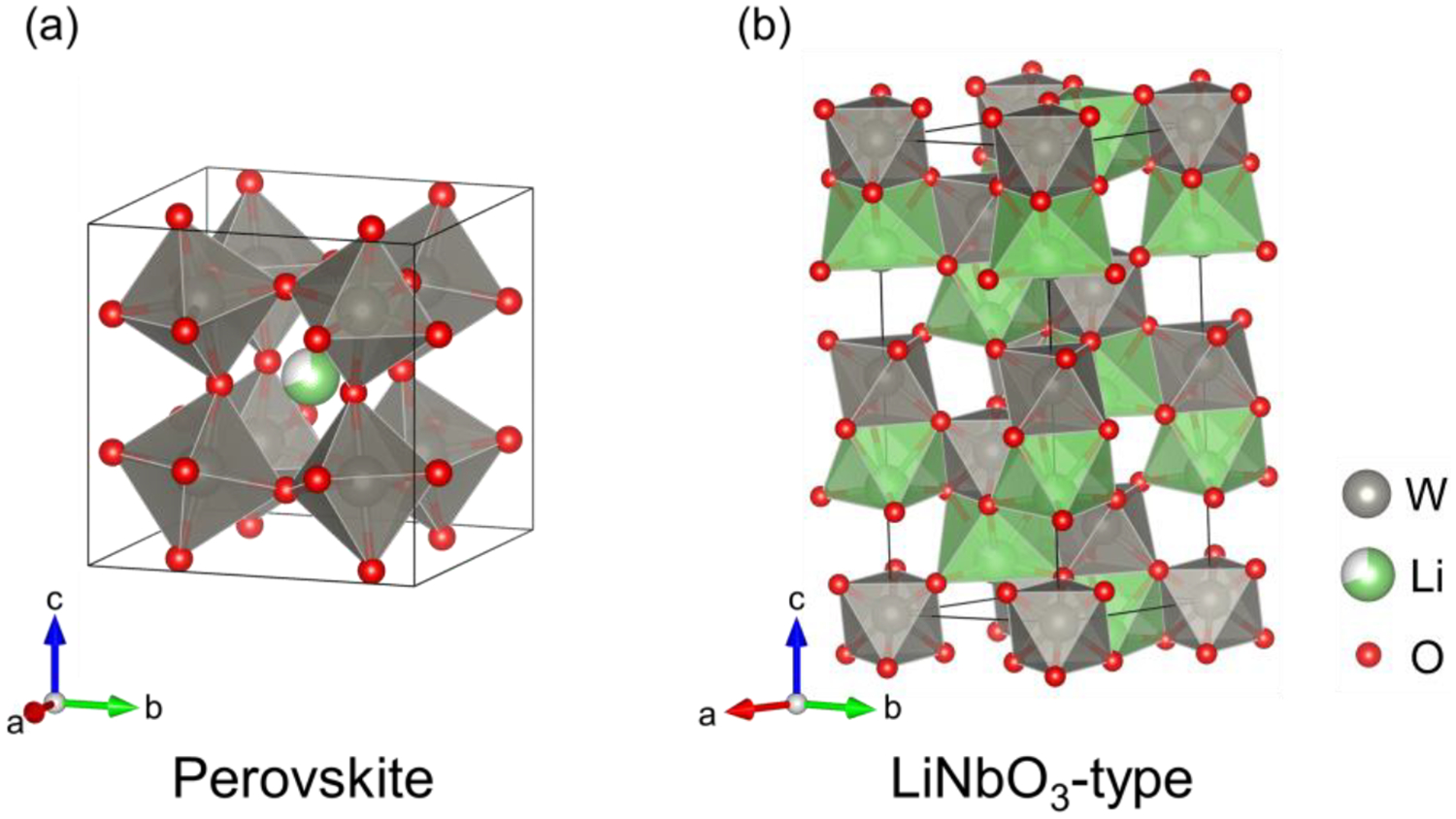
Crystal structures of Li_*x*_WO_3_ for (**a**) 0 ≤ *x* < 0.5 with the cubic perovskite (*Im*−3) structure reported previously [8] and (**b**) 0.5 ≤ *x* ≤ 1 with the LiNbO_3_-type (*R*3*c*) structure obtained in this work. The grey, green and red balls represent W, Li and O atoms, respectively. W- and Li-centered octahedra are shown in grey and green.

This study targeted lithium tungsten bronze, Li_*x*_WO_3_, which is known to exist in the compositional range of *x* ≤ 0.5 and has the perovskite (*Im*−3) structure, as shown in Figure 1a [8]. This phase has typically been prepared by a conventional high-temperature solid state reaction [9,10] and electrochemical reaction [11,12]. We report that the use of high pressure stabilizes the LiNbO_3_-type structure (Figure 1b), for the first time in the tungsten bronze family. In addition, Li_*x*_WO_3_ is *A*-site non-stoichiometric (0.5 ≤ *x* ≤ 1) with the variable *d* electron count of 0.5~1. We show the structural characterizations by means of X-ray diffraction (XRD) and neutron diffraction (ND), along with physical properties by electrical resistivity measurement.

## 2. Results and Discussion

### 2.1. Synthesis of Li_*x*_WO_3_ (0.5 ≤ *x* ≤ 1)

**Figure 2.**
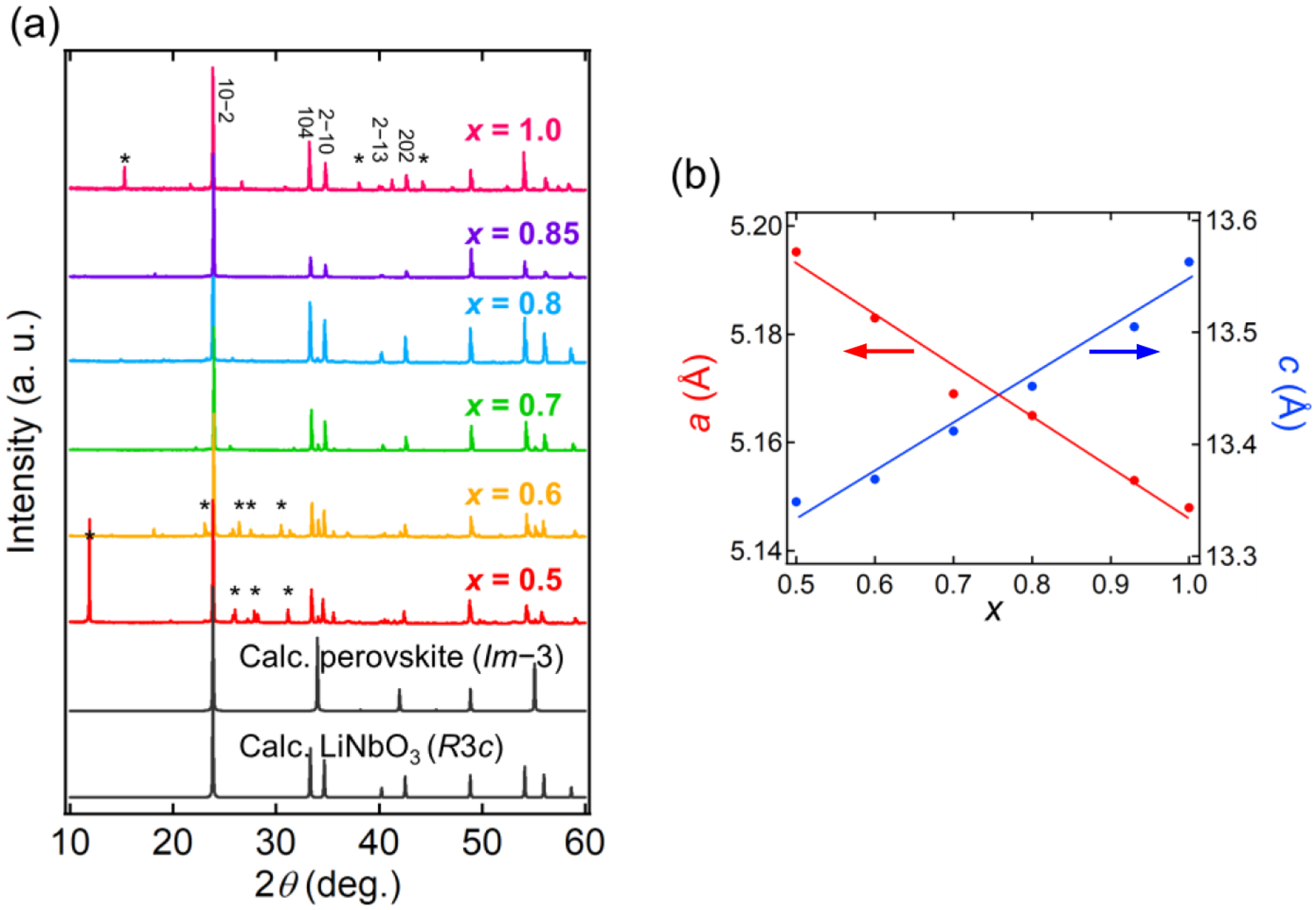
(**a**) XRD patterns of Li_*x*_WO_3_ (0.5 ≤ *x* ≤ 1.0) synthesized at 5–8 GPa, demonstrating the formation of the rhombohedral structure. Calculated patterns for the perovskite (*Im*−3) and LiNbO_3_ (*R*3*c*) structures are shown for comparison. Asterisks denote unreacted starting materials of Li_2_WO_4_, WO_2_, and WO_3_. (**b**) Lattice parameters, *a* (red) and *c* (blue), of the rhombohedral Li_*x*_WO_3_ phase as a function of *x*. The errors are within the size of the symbols. The solid lines are linear fits to the data.

Figure 2a shows the XRD patterns of Li_*x*_WO_3_ with 0.5 ≤ *x* ≤ 1. The diffraction patterns for all the samples are markedly different from that of the cubic (*Im*−3) perovskite for *x* < 0.5 [8] but are similar to that of LiNbO_3_ (Figure 2a). Single phases were obtained for *x* = 0.7, 0.8 and 0.85, while unreacted Li_2_WO_4_, WO_2_, and WO_3_ impurities were observed for the remaining samples. Modifying reaction temperature and pressure did not improve the results. Figure 2b shows the lattice parameters, *a* and *c*, as determined from Le Bail analysis. It is seen that the cell parameters evolve anisotropically as a function of *x*. When *x* is increased, the *a* and *c* axes decrease and increase, respectively. The linear evolution of both cell parameters, following the Vegard’s law, ensures the successful preparation of a solid solution for 0.5 ≤ *x* ≤ 1, though impurities were present for *x* ≤ 0.6 and *x* > 0.85. The difference in the density of the ambient-pressure phase of Li_0.5_WO_3_ and its high-pressure polymorph is very small (0.40%). The rhombohedral phase of Li_*x*_WO_3_ was extremely unstable at ambient temperature. Even in an inert atmosphere (e.g., in an Ar- or N_2_-flled glovebox), the sample starts to decompose within a few days into the known cubic (*Im*−3) perovskite of Li_*x*_WO_3_, possibly accompanied by Li extraction. The characterization of the materials was, therefore, carried out immediately after the syntheses, otherwise the samples were stored in liquid nitrogen.

### 2.2. Structural Characterizations of Li_0.8_WO_3_

**Figure 3.**
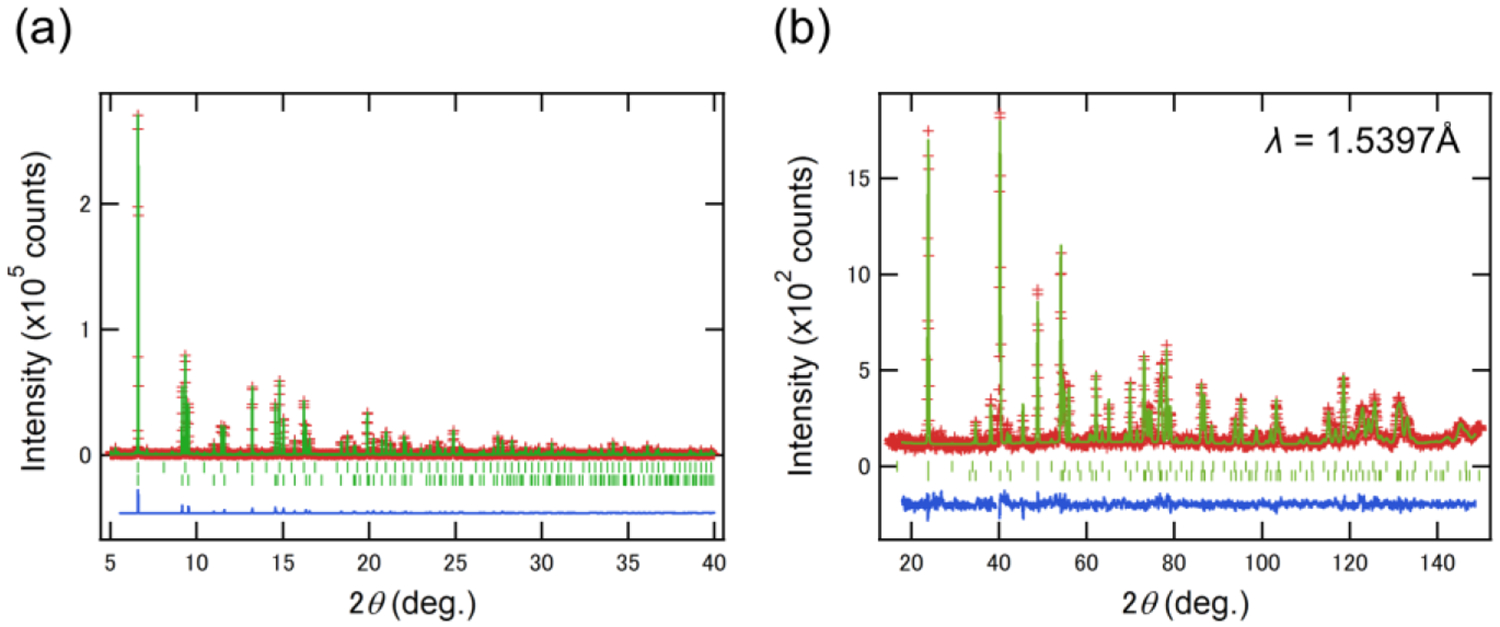
Rietveld refinement of (**a**) XRD and (**b**) ND data for Li_*x*_WO_3_ (*x* = 0.8) assuming the LiNbO_3_-type (*R*3*c*) structure. Red crosses, green solid line, and blue solid line represent observed, calculated, and difference intensities, respectively. The top and bottom green ticks indicate the positions of the Bragg peaks of the LiNbO_3_-type structure and the cubic (*Im*−3) phase with *x* ~ 0.5 (see text for details).

Extinction diffraction peaks in the XRD profiles suggest that the space group of the new phase is *R*3*c* or *R*−3*c*. We performed Rietveld refinement of the XRD data for the phase pure *x* = 0.8, assuming a non-centrosymmetric LiNbO_3_-type structure (*R*3*c*), as shown in Figure 3a. Li, W and O were placed at the 12*c* (0, 0, *z*), 6*b* (0, 0, 0) and 18*e* (*x*, *y*, *z*) site, respectively. Any Li deficiency was not taken into consideration at this stage due to low X-ray contrast for Li. The refined parameters are shown in Table 1. In order to examine the Li content, neutron measurements for *x* = 0.8 were conducted using the BT-1 powder diffractometer. Since the sample partially decomposed to the perovskite phase Li_*x*_WO_3_ (*x* < 0.5) with the *Im*−3 space group during shipping, the refinement was performed with inclusion of cubic-Li_0.5_WO_3_ (21.6(8)%) as a secondary phase, which resulted in goodness-of-fit parameters *R*_wp_ = 8.95%, *R*_p_ = 6.72%, and GOF = 1.16 and reasonable atomic parameters (Figure 3b and Table 1). The Li occupancy was estimated as 0.77(5), which is consistent with the nominal content of *x* = 0.8. The refinement of the 5 K data gave *R*_wp_ = 9.38%, *R*_p_ = 7.13%, and GOF = 1.41. We calculated an *A*O_6_ octahedral distortion parameter, Δ = 1/6Σ[(*d*_*i*_ − 〈*d*〉)/〈*d*〉]^2^, where *d*_*i*_ is the individual bond distance, and 〈*d*〉 is the average bond length [13]. We obtained Δ = 7.79 × 10^−3^ for Li_0.8_WO_3_. This value is larger than other +1/+5 type compounds (e.g., 4.27 × 10^−3^ for LiTaO_3_ [14] and 1.68 × 10^−3^ for LiNbO_3_ [15]) but is comparable with 8.15 × 10^−3^ for LiOsO_3_. The difference might be related to the *d* electron count. Refinement of the same neutron data with a *R*−3*c* model also gave similar reliability factors of *R*_wp_ = 8.95%, *R*_p_ = 6.72% and GOF = 1.16 (*R*_wp_ = 9.36%, *R*_p_ = 7.11% and GOF = 1.40 at 5 K). As such, we cannot completely rule out the possibility of a high-*T* LiNbO_3_ form with a centrosymmetric space group of *R*−3*c* without suitable single crystals. Note that the observed extinction reflections excluded the ilmenite-type structure (*R*−3).

### 2.3. Structural Transition in Li_x_WO_3_

Together with past research, this study has demonstrated the occurrence of compositional transition from the perovskite (*x* ≤ 0.5) to the LiNbO_3_-type structures (*x* ≥ 0.5). Given that the latter structure has a network composed of corner-sharing WO_6_ octahedra, one can then discuss this structural transition within the framework of perovskite chemistry. Using the Glazer notation, the observed compositional transition can be viewed as an octahedral tilting switching from three in-phase rotations (a^+^a^+^a^+^) for *x* ≤ 0.5 to three out-of-phase rotations (a^−^a^−^a^−^) for *x* ≥ 0.5, a transition which has not been observed in any perovskite-based materials. It is interesting to compare this system with the sodium, Na_*x*_WO_3_, where the a^+^a^+^a^+^ structure (*Im*−3) is stable over higher Na concentration up to the full stoichiometry (0.8 ≤ *x* ≤ 1) [7]. Our recent study on Na_*x*_WO_3_ revealed an unusual local phonon dynamic, which is interpreted as a rattling phenomenon based on loosely bound Na atoms at the 6*b* site with 12-fold coordination. This observation implies that the replacement of Na by smaller Li cations destabilizes the a^+^a^+^a^+^ structure. Li^+^ ions would be well suited to the six-fold coordination in the a^−^a^−^a^−^ structure (*x* ≥ 0.5).

### 2.4. Physical Properties

**Figure 4.**
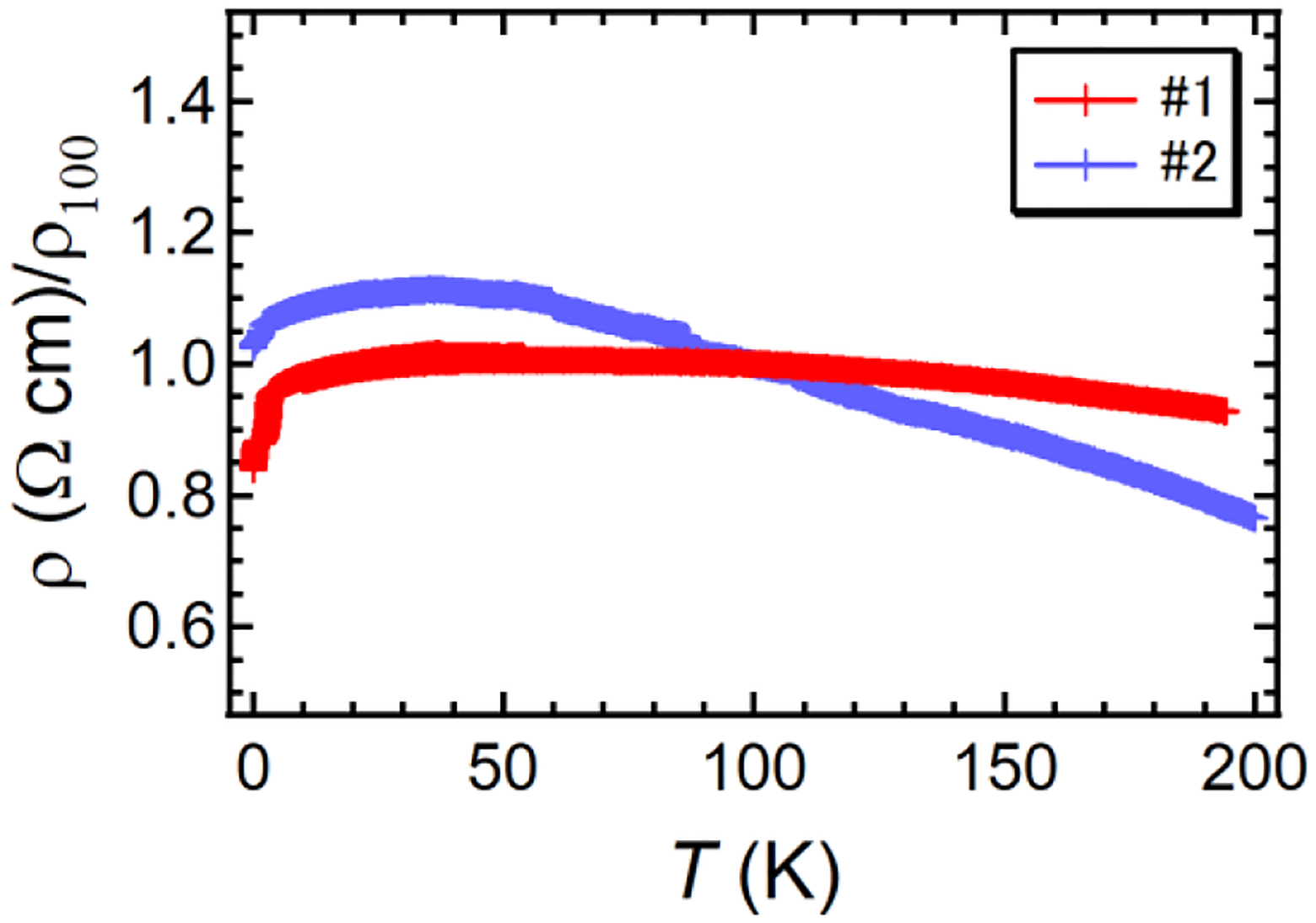
The temperature dependence of the electric resistivity of Li_0.8_WO_3_ normalized for the 100 K value. #1 and #2 are the batch numbers. *ρ*_100_ = 4.4 (#1) and 11 (#2) × 10^−2^ Ω cm.

The LiNbO_3_-type compounds are already successful in industrial applications, such as piezoelectric sensors and optical modulators, but the recent discovery of LiOsO_3_ opened a new avenue for the study of a “polar” metal, provoking many discussions on its mechanism [16–18]. What differentiates Li_*x*_WO_3_ (0.5 ≤ *x* ≤ 1) from LiOsO_3_ (d^3^) and MnTaO_2_N (d^5^) is the variable *d* electron count from 0.5 to 1.0, apart from a trace amount of defects in LiNbO_3_ (12*c* site) [19], thus giving a unique opportunity to tune and understand the physical properties of the LiNbO_3_-type structure. Unfortunately, the unstable nature of the samples and the presence of the impurity phases did not permit a systematic study. For this reason, we show hereafter the temperature dependence of the electrical resistivity (*ρ*) of Li_0.8_WO_3_, where two batches of samples (#1, #2) were used. As shown in Figure 4, both specimens show a metallic behavior in the low-temperature region below 40 K. However, we found a slightly negative temperature dependence (d*ρ*/d*T* < 0) above 40 K, which might be intrinsic but could be due to the effect of some grain boundary impedance. In addition, there is a drop below 3 K, which could be indicative of superconductivity, but the strong sample dependence and the absence of a peak in heat capacity measurements (not shown) indicate that this more likely arises from an impurity.

## 3. Materials and Methods

Polycrystalline samples of Li_*x*_WO_3_ (*x* = 0.5, 0.6, 0.7, 0.8, 0.85, 1.0) were prepared using a high-pressure (HP) technique. Stoichiometric mixtures of Li_2_WO_4_ (99%, Kojundo Chemical, Tokyo, Japan), WO_2_ (99%, Rare Metallic, Tokyo, Japan), and WO_3_ (99.999%, Rare Metallic, Tokyo, Japan) were ground in a mortar for 30–60 min and pressed into a pellet. Each pellet was sealed in a platinum capsule, inserted in a graphite tube heater, and enclosed in a pyrophyllite cube. These procedures were carried out in an N_2_-filled grove box. The pressure applied was 2 GPa for Li_0.5_WO_3_ and 5–8 GPa for a Li_*x*_WO_3_ (*x* > 0.5) using a cubic-anvil press, and the temperature was kept at 850–1200°C for 30 min during the reaction (see more detail in Table 2.). We observed a tendency that higher pressure (≥5 GPa) gives a higher purity phase.

Powder XRD patterns of polycrystalline samples of Li_*x*_WO_3_ were collected using an X-ray powder diffractometer (Bruker D8 Advance diffractometer, Cu *K*α), with the accelerating voltage and the applied current of 40 kV and 40 mA. Diffraction peaks were recorded in the 2*θ* range of 10–80° with a scan step of 0.2°·s^−1^. Powder neutron diffraction for Li_0.8_WO_3_ was collected at room temperature using a high-resolution powder diffractometer BT-1 at the National Institute of Standards and Technology (NIST) Center for Neutron Research (NCNR) (Gaithersburg, MD, USA). A Cu(311) monochromator was used to produce monochromatic neutron beams with a wavelength of 1.5397 Å. The powder sample was loaded into a vanadium cell. Rietveld refinement was performed using the JANA2006 package (Vaclav Petricek, Michal Dusek, and Lukas Palatinus, Prague, Czech Republic). Electrical resistivity of samples was collected using the Physical Properties Measurement System (PPMS, Quantum Design, San Diego, CA, USA) by the four-probe method using Au wire and Ag paste. The experimental setup was done in a glovebox right after the synthesis.

## 4. Conclusions

We have succeeded in expanding the Li concentration in the lithium tungsten bronze Li_*x*_WO_3_ using the high-pressure synthesis. Unlike the lower Li phase (0 < *x* ≤ 0.5) with the perovskite phase (*Im*−3), the new phase adopts the LiNbO_3_-type structure (*R*3*c*), notably with a variable A-site composition and thus electron count. The structural change can be regarded as the conversion of octahedral rotations from three in-phase rotations (a^+^a^+^a^+^) to three out-of-phase rotations (a^−^a^−^a^−^). Li_0.8_WO_3_ exhibits a metallic conductivity at low temperature. This study suggests a possibility that other LiNbO_3_-type compounds could also be susceptible to *A*-site deficiency, which may lead to exotic phenomena.

## Figures and Tables

**Table 1.** Structural parameters of Li_0.8_WO_3_ from Rietveld refinement on XRD and ND data at 300 K. *g* is the site occupancy factor. XRD: *a* = 5.1626(4), c = 13.4434(2) Å, *R*_wp_ = 12.43, *R*_p_ = 9.21% and GOF = 2.24. ND: *a* = 5.1665(3), c = 13.4424(10) Å, *R*_wp_ = 8.95, *R*_p_ = 6.72% and GOF = 1.16.

Technique	Atom	Wyckoff Position	*g*	*x*	*y*	*z*	*U*_iso_ (Å^2^)
XRD	Li	12*c*	1	0	0	0.2502(6)	0
	W	6*b*	1	0	0	0	0
	O	18*e*	1	0.076(3)	0.371(6)	0.0829(2)	0
ND	Li	12*c*	0.77(5)	0	0	0.2748(16)	0.014(6)
	W	6*b*	1	0	0	0	0.0034(8)
	O	18*e*	1	0.0659(12)	0.332(2)	0.0821(14)	0.0063(5)

**Table 2.** Synthesis conditions of Li_*x*_WO_3_.

*x*	0.5	0.6	0.7	0.8	0.85	1.0
**Pressure (GPa)**	2	5	5	6	6	8
**Temperature (°C)**	850	850	1000	1200	1200	1200
